# Supplementary material for: SIMPEL: using stable isotopes to elucidate dynamics of context specific metabolism
Source: Commun Biol. 2024 Feb 12;7:172. doi: 10.1038/s42003-024-05844-z (PMC10861564; doi:10.1038/s42003-024-05844-z)
Supplement: Supplementary file 2 — Supplementary Information [file 42003_2024_5844_MOESM2_ESM.pdf]

Supplementary information:

SIMPEL: using stable isotopes to elucidate dynamics of context specific metabolism

Shrikaar Kambhampati[1†*⊥], Allen Hubbard[1†], Somnath Koley[1†], Javier D. Gomez[2], Frédéric Marsolais[3, 4], Bradley S. Evans[1], Jamey D. Young[2, 5], Doug K. Allen[1, 6*]

[1]Donald Danforth Plant Science Center, St. Louis, MO, USA 63141; [2]Chemical and Biomolecular Engineering, Vanderbilt University, Nashville, TN, USA 37235; [3]London Research and Development Center, London, Ontario, Canada N5V 4T3; [4]Department of Biology, University of Western Ontario, London, Ontario Canada N6A 5B7, [5]Department of Molecular Physiology and Biophysics, Vanderbilt University, Nashville, TN, USA 37235, [6]Agricultural Research Service, US Department of Agriculture, St. Louis, MO, USA 63141.

*Corresponding authors: Shrikaar Kambhampati, skambhampati@salk.edu; Doug K Allen, doug.allen@ars.usda.gov

[†]These authors contributed equally to the work

[⊥]Present address: Salk Institute of Biological Studies, La Jolla, CA, USA 92127

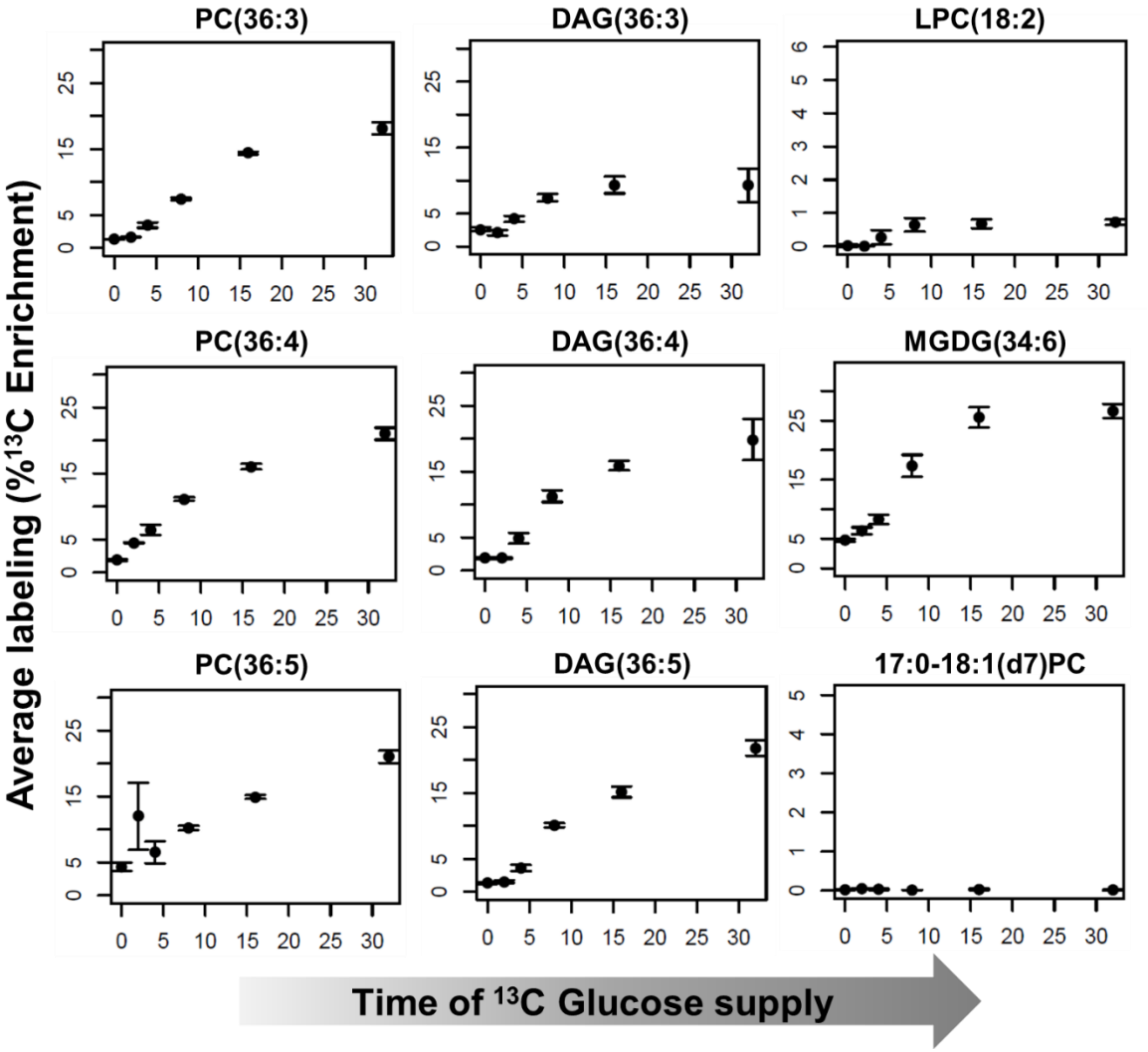

Supplementary fig S1: Average labeling images for all lipids were automatically generated using SIMPEL and exported as pdfs. A set of representative lipids relevant to the description provided in main text along with an example standard (control) that was spiked in before extraction (17:0-18:1(d7)PC) as part of the ultimate splash one lipidomix, are presented here.

Supplementary text T1: Determining the HRMS resolution for data acquisition from isotope enriched metabolomics experiments.

The instrument resolution required to differentiate minor differences in masses can be calculated using the formula R = M / ΔM, where M is the molecular ion (m/z) that needs to be resolved and ΔM is the difference in m/z between the ions that need to be distinguished. For example, to distinguish PC (36:4) (m/z = 782.5694 Da) from $^{13}C_2$ labeled PC (36:5) (m/z = 782.5602), each of these signals should have a FWHM peak width of approximately 0.009/2 Da. This requires a resolution of 782/(0.009/2) = ~175,000. Typically, orbitrap instrument resolution capability is represented for an m/z of 200 Da. Since resolution is inversely proportional to the square root of the m/z and proportional to acquisition time, a higher resolution setting (FWHM ~250,000) is required within the acquisition method to achieve >175,000 at m/z >782 Da, depending on the type of instrument being used. More details on determining the instrument resolution to distinguish overlapping signals at desired m/z can be found in Makarov et al., 2009[1].

In most situations, however, as is the case with the current manuscript, C8 chromatography can be used to resolve PC molecular species with different desaturation units. This alleviates the need for such high resolution. If labeling within the individual fatty acid chains of lipid molecular species is desired, as highlighted in this manuscript, the need for HRMS at the MS2 level still exists, as the 18:2 (m/z = 279.2324) and $^{13}C_2$ of 18:3 (m/z = 279.2240) within PC (36:5), for example, still needs to be distinguished. This requires a resolution, R = 279/(0.008/2) =~70000 FWHM to sufficiently differentiate the labeled isotopologues. Users are encouraged to determine the required resolution prior to data acquisition, to adequately identify isotopologues from the labeling experiment weather it is a dual labeled, or single labeled experiment.

Reference:

1. Makarov, A., Denisov, E. & Lange, O. Performance evaluation of a high-field orbitrap mass analyzer. *J Am Soc Mass Spectrom* **20**, 1391–1396 (2009).
