# Supplementary material for: SIMPEL: using stable isotopes to elucidate dynamics of context specific metabolism
Source: Commun Biol. 2024 Feb 12;7:172. doi: 10.1038/s42003-024-05844-z (PMC10861564; doi:10.1038/s42003-024-05844-z)
Supplement: Supplementary file 3 — Description of Supplementary Materials [file 42003_2024_5844_MOESM3_ESM.docx]

**Description of Additional Supplementary Files**

**File name:** Supplementary Data 1

**Description:** The source data behind the figures.

**File name:** Supplementary Data 2

**Description:** matlab file containing flux model for 13C only flux map

**File name:** Supplementary Data 3

**Description:** matlab file containing flux model for 13C15N dual label flux map

**File name:** Supplementary Data 4

**Description**: Matlab file containing flux model for 13C and 15N parallel labeling flux map
