# Supplementary material for: SIMPEL: using stable isotopes to elucidate dynamics of context specific metabolism
Source: Commun Biol. 2024 Feb 12;7:172. doi: 10.1038/s42003-024-05844-z (PMC10861564; doi:10.1038/s42003-024-05844-z)
Supplement: Supplementary file 8 — Reporting Summary [file 42003_2024_5844_MOESM8_ESM.pdf]

nature research

Corresponding author(s): Doug Allen & Shrikaar Kambhampati

Last updated by author(s): Jan 5, 2024

Reporting Summary

Nature Research wishes to improve the reproducibility of the work that we publish. This form provides structure for consistency and transparency in reporting. For further information on Nature Research policies, see our Editorial Policies and the Editorial Policy Checklist.

Statistics

For all statistical analyses, confirm that the following items are present in the figure legend, table legend, main text, or Methods section.

n/a	Confirmed	
☐	☒	The exact sample size (n) for each experimental group/condition, given as a discrete number and unit of measurement
☐	☒	A statement on whether measurements were taken from distinct samples or whether the same sample was measured repeatedly
☐	☒	The statistical test(s) used AND whether they are one- or two-sided<br>*Only common tests should be described solely by name; describe more complex techniques in the Methods section.*
☐	☒	A description of all covariates tested
☐	☒	A description of any assumptions or corrections, such as tests of normality and adjustment for multiple comparisons
☐	☒	A full description of the statistical parameters including central tendency (e.g. means) or other basic estimates (e.g. regression coefficient) AND variation (e.g. standard deviation) or associated estimates of uncertainty (e.g. confidence intervals)
☐	☒	For null hypothesis testing, the test statistic (e.g. F, t, r) with confidence intervals, effect sizes, degrees of freedom and P value noted<br>*Give P values as exact values whenever suitable.*
☒	☐	For Bayesian analysis, information on the choice of priors and Markov chain Monte Carlo settings
☒	☐	For hierarchical and complex designs, identification of the appropriate level for tests and full reporting of outcomes
☒	☐	Estimates of effect sizes (e.g. Cohen's d, Pearson's r), indicating how they were calculated

Our web collection on statistics for biologists contains articles on many of the points above.

Software and code

Policy information about availability of computer code

Data collection	Thermofisher Scientific, XCalibur(TM) Instrument Control Software (Commercial) was used for all Mass Spectrometry data acquisition.
Data analysis	Custom code generated for the tool SIMPEL are deposited in Github. Opensource tools XCMS and MZmine were used for preprocessing of MS datasets. Commertial software XCalibur (qualbrowser) and Compound Discoverer were used for data visualization, quanitification of MS2 and also for Compound identification. Open source package IsoCorrectoR was used for natural abundance correction, and was integrated into SIMPEL's workflow. Commercial tool INCA was used for Metabolic Flux Analysis.

For manuscripts utilizing custom algorithms or software that are central to the research but not yet described in published literature, software must be made available to editors and reviewers. We strongly encourage code deposition in a community repository (e.g. GitHub). See the Nature Research guidelines for submitting code & software for further information.

Data

Policy information about availability of data

All manuscripts must include a data availability statement. This statement should provide the following information, where applicable:

- Accession codes, unique identifiers, or web links for publicly available datasets
- A list of figures that have associated raw data
- A description of any restrictions on data availability

Raw and pre-processed data for stable isotope labeled metabolomics using Arabidopsis roots and lipidomics using Camelina seeds is available at the NIH Common Fund's National Metabolomics Data Respository (NMDR) website, the Metabolomics Workbench, https://www.metabolomicsworkbench.org/, where they have been assigned study IDs ST002240 and ST002239 respectively. The data for metabolomics and lipidomics experiments can be accessed directly via their project (PR001429) doi:http://dx.doi.org/10.21228/M80X3B.
The tool SIMPEL is freely available for public use at https://github.com/SIMPELmetabolism/SIMPEL, as an R package along with test data and source code. The tool

is also available at the metabolomics workbench at https://www.metabolomicsworkbench.org/data/simpel_load.php, as a web tool. A tutorial for SIMPEL usage with the test data is provided as supplemental file 3, with this article.

Field-specific reporting

Please select the one below that is the best fit for your research. If you are not sure, read the appropriate sections before making your selection.

☒ Life sciences ☐ Behavioural & social sciences ☐ Ecological, evolutionary & environmental sciences

For a reference copy of the document with all sections, see nature.com/documents/nr-reporting-summary-flat.pdf

Life sciences study design

All studies must disclose on these points even when the disclosure is negative.

Sample size	Three (lipid labeling) or Four (dual-labeling experiment) replicates were used for time course pulse labeling experiments. Since no direct comparisons from one time point to another was made with descriptive statistics, we reasoned that this sample size would suffice. The entire time course label enrichment data was used to compare between compounds for k-means clustering analysis, which did consider standard deviation between all data points within the time course, providing sufficient information on variability. Sample size used for flux analyses was also considered acceptable based on prior publications from scientific community using these analyses. For MS2 data analyses, an average intensity of four scans were used for each labeled fatty acid fragment. No claims on significance using a statistical test were made, rather we used the trends in labeling between time points to deduce conclusions.
Data exclusions	No data were excluded from planned studies
Replication	All experiments for which biological findings were reported were repeated atleast twice. Best parameters for data acquisition and instrument control were determined using previous publications and optimization using commercial standards, where available. Data preprocessing for metabolomics datasets were perfomed using parameters that were optimized using a design of experiments (DoE) strategy within Isotopologue Parameter Optimization (IPO) open source software package. The software tool presented in this manuscript was evaluated with a minimum of five different datasets (two of which we present with biological findings) to ensure sufficient reproducibility for each of the functions made available.
Randomization	For arabidopsis root labeling experiments, each replicate contained root tissue collected from ~50 arabidopsis seedlings in a culture plate. Four of these replicates were grouped as an experimental variable. Similarly for Camelina seed labeling experiments, all seeds from 4 pods picked randomly from plants were excised and used as a single replicate. In both cases, we reasoned that an adequate representation of heterogeneity was captured per replicate within each sample group. For data acquisition, samples queues were set up to collect from lowest labeling time point (unlabeled) to highest time point, to avoid labeled compound carry over from sample to sample, and were not randomized. We did, however, test and determined that the chromatographic carry over was $<0.1\%$ for select set of representative compound standards throughout the chromatographic run. Since, the experiment involves a metabolomics type data analysis approach, data analysis approach we decided not to take chances with carry over/contatimation of labeled compounds from sample to sample.
Blinding	Since each replicate within a sample group included adequate representation of heterogeneity from several individual seedlings/pods (see sample grouping above), we reasoned that investigator blinding was not necessary.

Reporting for specific materials, systems and methods

We require information from authors about some types of materials, experimental systems and methods used in many studies. Here, indicate whether each material, system or method listed is relevant to your study. If you are not sure if a list item applies to your research, read the appropriate section before selecting a response.

Materials & experimental systems

n/a	Involved in the study	
☒	☐	Antibodies
☒	☐	Eukaryotic cell lines
☒	☐	Palaeontology and archaeology
☒	☐	Animals and other organisms
☒	☐	Human research participants
☒	☐	Clinical data
☒	☐	Dual use research of concern

Methods

n/a	Involved in the study	
☒	☐	ChIP-seq
☒	☐	Flow cytometry
☒	☐	MRI-based neuroimaging
